# Socioeconomic status and the incidence of non-central nervous system childhood embryonic tumours in Brazil

**DOI:** 10.1186/1471-2407-11-160

**Published:** 2011-05-05

**Authors:** Beatriz de Camargo, Juliana Moreira de Oliveira Ferreira, Rejane de Souza Reis, Sima Ferman, Marceli de Oliveira Santos, Maria S Pombo-de-Oliveira

**Affiliations:** 1 Pediatric Hematology and Oncology Program, Research Center, Instituto Nacional de Câncer, Rio de Janeiro, Brazil; Rua Andre Cavalcanti, 37, CEP 20231-050, Rio de Janeiro, Brazil; 2 Coordenação de Prevenção e Vigilância, Instituto Nacional do Câncer, Rio de Janeiro, Rua dos Inválidos, 212, 3rd floor, CEP 20231-048, Brazil; 3 Pediatric Department, Instituto Nacional de Câncer, Rio de Janeiro, Brazil, Praça Cruz Vermelha, Rio de Janeiro, Brazil

## Abstract

**Background:**

Childhood cancer differs from most common adult cancers, suggesting a distinct aetiology for some types of childhood cancer. Our objective in this study was to test the difference in incidence rates of 4 non-CNS embryonic tumours and their correlation with socioeconomic status (SES) in Brazil.

**Methods:**

Data was obtained from 13 Brazilian population-based cancer registries (PBCRs) of neuroblastoma (NB), Wilms'tumour (WT), retinoblastoma (RB), and hepatoblastoma (HB). Incidence rates by tumour type, age, and gender were calculated per one million children. Correlations between social exclusion index (SEI) as an indicator of socioeconomic status (SES) and incidence rates was investigated using the Spearman's test.

**Results:**

WT, RB, and HB presented with the highest age-adjusted incidence rates (AAIRs) in 1 to 4 year old of both genders, whereas NB presented the highest AAIR in ≤11 month-olds. However, differences in the incidence rates among PBCRs were observed. Higher incidence rates were found for WT and RB, whereas lower incidence rates were observed for NB. Higher SEI was correlated with higher incidences of NB (0.731; p = 0.0117), whereas no SEI correlation was observed between incidence rates for WT, RB, and HB. In two Brazilian cities, the incidence rates of NB and RB were directly correlated with SEI; NB had the highest incidence rates (14.2, 95% CI, 8.6-19.7), and RB the lowest (3.5, 95% CI, 0.7-6.3) in Curitiba (SEI, 0.730). In Natal (SEI, 0.595), we observed just the opposite; the highest incidence rate was for RB and the lowest was for NB (4.6, 95% CI, 0.1-9.1).

**Conclusion:**

Regional variations of SES and the incidence of embryonal tumours were observed, particularly incidence rates for NB and RB. Further studies are necessary to investigate risk factors for embryonic tumours in Brazil.

## Background

Childhood cancer differs from most common adult cancers, suggesting a distinct aetiology for some types of childhood cancer. Embryonic tumours that afflict very young children probably originate from immature tissue, and their microscopic morphological appearance resembles tissues in the developing embryo and foetus. Defects in tissue growth pathways and in their differentiation during the prenatal/postnatal period would promote tumour genesis [1,2].

Age standardized incidence rates for neuroblastoma (NB), Wilms' tumour (WT), retinoblastoma (RB), and hepatoblastoma (HB) vary worldwide from 7-12, 4-10, 3-5, and 0.8-1 per million children less than 15 years of age, respectively [3]. Lower incidence rates of NB were observed in southern and eastern Asia and were extremely rare in African countries, but had higher incidence rates in Western European countries, the United States, Australia, and New Zealand [4]. Regarding WT, higher incidence rates were observed in Sweden, Afro-Americans, and in São Paulo, Brazil [3]. In developing countries, the incidence rates of RB are generally higher and seem to be associated with non-inheritable forms of the disease. These variations in incidence, which may be related to possible risk factors and geographical and ethnic patterns, provide clues to aetiological factors. Additionally, socioeconomic status (SES) might have implications and/or associations with the different incidence rates [3-5]. Residential area and parental educational attainment level were used as indicators of SES, and the risk of childhood cancers was tested mostly in acute leukaemia, but few studies also analyzed embryonal tumours [6-8]. Although some evidence exists for an association, it is not conclusive. The possible association between SES and childhood cancer risk have raised the hypothesis that potential aetiological factors could be linked with modern lifestyles.

There are a lack of studies regarding the regional differences in the incidence of embryonic tumours such as WT, RB and NB; consequently, there are very limited data on causes and pathologic mechanistic pathways. RB has a genetic aetiology due to somatic or germinal RB1 gene mutations [9]. The International Agency for Research on Cancer (IARC) has promoted multicenter studies to uncover the occurrence of childhood cancer to provide etiological clues. In the first descriptive analysis of the incidence of childhood cancer in Brazil, higher age-specific incidence rates of RB were observed in some areas of the country [10]. Our objective in the study was to test whether SES correlated with the incidence rates of WT, RB, NB and HB as the main non-central nervous system embryonic tumours. The social exclusion index (SEI) was used because it is a synthesis of all variables related to poverty and inequalities. This index was constructed based on suitable life conditions, literacy, and youth vulnerability similar to that applied for the Human Development Index [11,12].

## Methods

### Dataset

Data analysed were extracted from the databases of PBCRs located in major Brazilian cities as described previously [10]. For this analysis, 13 of 14 PBCRs were selected; one PBCR (Jaú) was excluded because no embryonic tumours were registered. Children's age ranges were placed into four groups as follows: younger than 12 months of age, ≥1 to 4 years of age; 5 to 9 years of age; and 10 to ≤14 years of age. The International Classification of Childhood Cancer was used and only the following tumours were selected: NB and ganglioneuroblastoma (from Group IV the subgroup IVa), RB (Group V), WT (from Group VI- the subgroup VI.a), and HB (from Group VII the subgroup VIIa) [13]. The cumulative records of RB cases from the PBCR database did not classify RB as bilateral and unilateral. Ethical approval was waived for the kind of study.

### Social exclusion index

The description of the SEI construction is described in detail elsewhere [11]. Briefly, the first dimension includes a poverty indicator, an employment indicator, and an inequality indicator; the second dimension comprises 2 indicators of literacy and parental years of study; and the last dimension includes a youth and a regional violence indicator. Cities were classified by the social exclusion index (SEI), which ranges from 0 (minimum) to 1 (maximum) with a higher SEI score indicating better socio-economic status.

### Statistical methods

Age-adjusted incidence rates (AAIR) were estimated with the direct method using the world population proposed for age groups younger than 15 years [14]. The incidence rate per 1,000,000 inhabitants refers to the risk of new cases. For all 13 PBCRs included, analyses included the number of new cases, the absolute and relative values, and AAIR according to tumour type, gender, and age range.

Correlations between SEI and incidence rates were investigated using Spearman's test. The 95% confidence intervals (CI) were calculated using the Poisson approximation, or exactly when less than 30 cases were observed.

## Results

AAIR for each of the selected embryonic tumours are shown in Table 1. The median AAIR of NB in both sexes was 5.9 per million, and was lower in Manaus (2.3, 95% CI, 0.0-4.6) and higher in Curitiba (14.2, 95% CI, 8.6-19.7). The median AAIR of WT was 9.5 per million, and was lower in Natal (5.2, 95% CI, 0.0-10.3) and higher in Goiania (18.0, 95% CI, 10.6-25.4). The median AAIR of RB was 6.6 per million, and was lower in Curitiba (3.5, 95% CI, 0.7-6.3) and higher in Natal (12.7, 95% CI, 4.3-21.0). The median AAIR of HB varied from 0.0 to 2.8 per million as shown in Table 1.

**Table 1:** Age adjusted incidence rate (AAIR) per million for embryonal tumours in 13 Brazilian PBCR

PBCR	AAIR* (0 to 14 years)							
	NB	95% CI	RB	95% CI	WT	95% CI	HB	95% CI
Aracaju (1996-2000)	5.90	(-0.78;12.58)	6.99	(0.09;13.90)	10.93	(2.13;19.72)	1.97	(-3.45;7.39)
Campinas (1991-1995)	5.63	(11.11;10.16)	7.58	(2.31;12.85)	9.53	(3.61;15.45)	1.52	(-1.43;4.46)
Curitiba (1998 - 2002)	14.18	(8.60;19.75)	3.52	(0.70;6.34)	5.76	(2.19;9.34)	0.00	-
Distrito Federal (1999-2002)	7.13	(3.63;10.64)	3.80	(1.17;6.44)	10.47	(6.18;14.77)	0.48	(-0.83;1.78)
Fortaleza (1998-2002)	2.40	(0.61;4.19)	3.55	(1.35;5.76)	6.37	(3.42;9.32)	0.00	-
João Pessoa (2000-2004)	3.54	(-0.52;7.60)	4.32	(-0.57;9.20)	12.37	(4.26;20.49)	0.00	-
Manaus (1999-2002)	2.33	(0.05;4.61)	5.83	(2.22;9.44)	6.29	(2.57;10.01)	1.59	(-0.96;4.14)
Natal (1998-2001)	4.60	(0.08;9.12)	12.67	(4.33;21.02)	5.18	(0.03;10.33)	0.00	-
Porto Alegre (1998-2002)	11.82	(6.16;17.48)	7.54	(2.87;12.21)	13.56	(7.43;19.69)	2.78	(-1.08;6.64)
Recife (1997-2001)	11.18	(5.98;16.38)	4.98	(1.51;8.45)	13.48	(7.92;19.04)	0.43	(-0.75;1.60)
Salvador (1998-2002)	4.91	(2.24;7.57)	6.59	(3.54;9.65)	9.48	(5.83;13.13)	2.13	(-0.27;4.53)
Goiânia (1999-2003)	11.51	(5.65;17.37)	8.34	(3.17;13.51)	18.03	(10.63;25.44)	0.00	-
São Paulo (1998-2002)	9.60	(7.80;11.41)	9.08	(7.32;10.85)	8.52	(6.84;10.19)	0.80	(0.07;1.54)

**Median**	**5.90**		**6.59**		**9.48**		**0.43**	

* per million

Abbreviations: NB. Neuroblastoma; RB. Retinoblastoma; WT. Wilms' tumors; HB. Hepatoblastoma;

Source: PBCR: Population-Based Cancer Registries; MP/Fundação Instituto Brasileiro de Geografia e Estatística (IBGE); MS/INCA/CONPREV/Information Division. 2008 (http://www.inca.gov.br).

The age specific incidence rates (ASIR) are shown in Table 2. The higher ASIR of NB was seen in children ≤11 months of age in the majority of the PBCR, whereas in two regions (Curitiba and Distrito Federal) a higher ASIR for NB was found in children ≥1 to 4 years of age. WT had the highest ASIR in children between the ages of ≥1 to 4 years with a median of 18.7 cases per million. RB had the highest ASIR in children between the ages of ≥1 to 4 years with a median of 15.6 cases per million.

**Table 2:** Age-standardized incidence rate (ASIR) per million for embryonal tumours in 13 Brazilian PBCR

	ASIR															
	
PBCR	0 to 11 months				1 to 4 years				5 to 9 years				10 to 14 years			
	
	NB	RB	WT	HB	NB	RB	WT	HB	NB	RB	WT	HB	NB	RB	WT	HB
**Aracaju (1996-2000)**	25.01	0.00	0.00	25.01	12.76	12.76	25.51	0.00	0.00	9.48	9.48	0.00	0.00	0.00	0.00	0.00
**Campinas (1991-1995)**	26.30	26.30	39.45	0.00	9.34	15.57	18.69	0.00	2.35	2.35	2.35	0.00	0.00	0.00	0.00	0.00
**Curitiba (1998 - 2002)**	15.28	7.64	7.64	0.00	35.98	9.47	15.15	0.00	4.44	0.00	1.48	0.00	1.40	0.00	0.00	0.00
**Distrito Federal (1999-2002)**	5.99	0.00	5.99	0.00	12.36	12.36	24.72	1.55	7.72	0.00	5.15	0.00	1.26	0.00	2.51	0.00
**Fortaleza (1998-2002)**	0.00	9.65	4.82	0.00	5.91	7.10	15.38	0.00	0.95	1.89	3.79	0.00	0.89	0.00	0.00	0.00
**João Pessoa (2000-2004)**	0.00	0.00	18.65	0.00	4.64	13.92	27.85	0.00	3.57	0.00	7.14	0.00	3.26	0.00	0.00	0.00
**Manaus (1999-2002)**	7.38	7.38	0.00	0.00	5.67	17.01	17.01	1.89	0.00	0.00	3.25	1.66	0.00	0.00	0.00	1.66
**Natal (1998-2001)**	0.00	38.77	19.38	0.00	0.00	24.17	4.83	0.00	11.21	3.74	3.74	0.00	3.38	3.38	3.38	0.00
**Porto Alegre (1998-2002)**	19.07	9.53	9.53	9.53	22.02	22.02	31.80	4.89	7.72	0.00	7.72	1.79	3.58	0.00	1.79	1.79
**Recife (1997-2001)**	25.96	17.31	0.00	0.00	23.26	8.46	25.38	0.00	4.80	3.20	16.00	1.47	1.47	0.00	1.47	1.47
**Salvador (1998-2002)**	24.74	9.89	4.95	4.95	9.72	15.79	24.29	3.64	0.00	2.89	4.82	0.00	0.00	0.00	0.00	0.00
**Goiânia (1999-2003)**	21.74	32.61	43.47	0.00	18.82	18.82	32.26	0.00	10.63	0.00	12.76	0.00	1.96	0.00	1.96	0.00
**São Paulo (1998-2002)**	22.14	24.47	10.49	3.50	20.26	20.85	16.45	1.47	3.81	1.67	6.91	0.00	1.32	0.66	1.32	0.00

**Median**	**15.28**	**9.53**	**5.99**	**0.00**	**12.36**	**15.57**	**18.69**	**0.00**	**3.81**	**0.00**	**4.82**	**0.00**	**1.32**	**0.00**	**0.00**	**0.00**

Abbreviations: NB. Neuroblastoma; RB. Retinoblastoma; WT. Wilms' tumours; HB. Hepatoblastoma; Source: Population-Based Cancer MP/Fundação Instituto Brasileiro de Geografia e Estatística (IBGE); MS/INCA/CONPREV/Information Division. 2008 (www.inca.gov.br).

**Figure 1:**
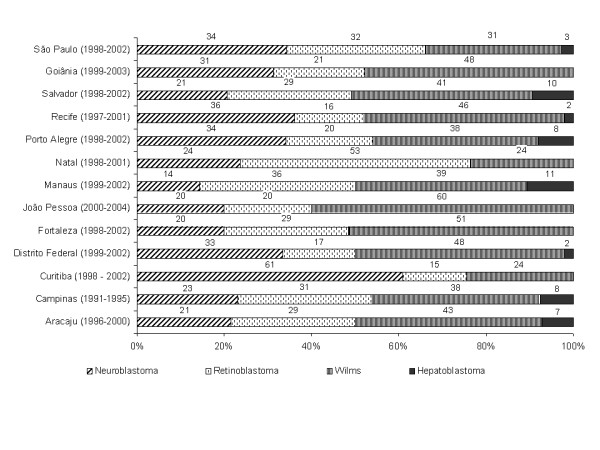
**Percentage distribution of NB, WT, RB, HB, expressed as the total cases of all 4 embryonal tumours**.

The ratio distribution of the 4 selected embryonic tumours is shown in Figure 1. The most common tumour in 10 PBTRs in the percentage distribution was WT (38-60%), whereas NB was the most common in only two PBCRs in the percentage distribution; São Paulo (34%) and Curitiba (61%). RB was the most common embryonic tumour in Natal (53%).

**Figure 2:**
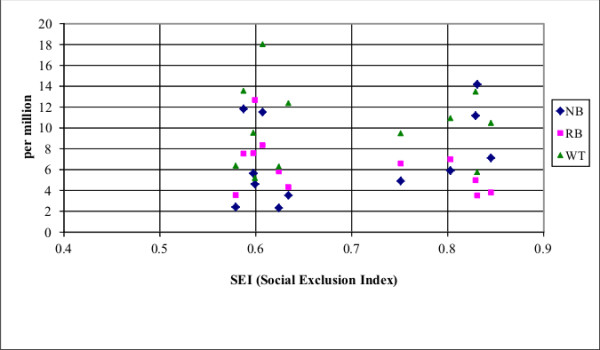
**Correlation between the SEI and AAIR for 3 embryonal tumours (NB, WT, RB)**.

The SEI varied from 0.522 to 0.761. The lowest SEI was observed in the city of Manaus located in the north (0.522) and the highest SEI was observed in Porto Alegre (0.761) in southern Brazil. The correlation between SEI and the incidence rates for the 3 embryonic tumours (NB, RB, WT) are shown in Figure 2. Overall, the correlation between SEI and WT (p = 0.4865), RB (p = 0.6496), and HB (p = 0.5843) were not statistically significant (Spearman's rank correlation test), but the incidence rate of NB was significantly correlated with SEI (0.731; p = 0.0117). The differences in the incidence rates of NB and RB correlating with SEI were observed in Curitiba and Natal. In Curitiba (SEI, 0.730), a high AAIR of NB and the lowest AAIR of RB was observed, whereas the opposite was observed in Natal (SEI, 0.595) with a high AAIR of RB and one of the lowest AAIR of NB.

## Discussion

Incidence rates, age, and gender distributions provide insights into tumour initiation and aetiology. This is the first report that explores the geographic variations in the incidence of select embryonic tumours and differences in SES in an emerging middle-income country like Brazil.

NB has the fourth highest incidence rate in children less than 14 years of age in developed countries [15,16]. In a recent analysis of the Surveillance, Epidemiology, and End Results database, the incidence of NB seems to be increasing in nonmetropolitan counties of the United States [17]. A very low incidence rate was observed in Mexico, even in children under one year of age [18,19]. Historically, the incidence rates of NB are generally higher in regions with a higher standard of living [20]. In 13 Brazilian PBCRs, the NB incidence rates varied from 2.3 to 14.2 per million. The higher incidence correlated with Brazilian cities with high SEIs. Additionally, we observed that the higher incidence rates occurred in children between the ages of 1 to 4 years living in cities with high SEIs (Curitiba, DF and Porto Alegre). These data might corroborate the previous speculation related to the timing of the diagnosis [21]. The biology of NB cases includes spontaneous regression in children less than one year of age. The ASIR differences observed in the cities independently of the SEI ruled out that under-diagnosis or under-ascertainment of NB cases as the reasons for distinct incidence rates in Brazilian regions. Etiological studies have explored the environmental and genetic predisposition of NB [22].

WT incidence rates world-wide are lower (4 to 10 cases per million) than those of NB according to data reported by IARC [4]. The rate of WT is higher in females than males and no significant changes in incidence rates were reported. In São Paulo, the WT incidence rates have remained stable (8.5 per million) since the 1990s [3,4]. Presently, we observed higher incidence rates in 8 PBCR, which vary from 9.5 per million in Salvador to 18 per million children in Goiania. WT was the most common embryonic tumour in 10 of the 13 PBCRs analysed. Higher incidences rates were observed in children between 1 and 4 years of age. SEI was not related to the incidence rates of WT. For instance, high WT incidence rates were observed in Porto Alegre (SEI, 0.761) and João Pessoa (SEI, 0.559), whereas low incidence rates were found in Natal (SEI, 0.595) and Curitiba (SEI, 0.730). The potential involvement of genetic and environment exposures in the development of WT is challenge to be explored, because in WT both genetic and environmental component are involved, although the exact aetiology remains unclear [23,24]. In a small case-control study conducted in different Brazilian cities, positive associations among parental exposure to pesticides before birth and maternal exposure to dipyrone during pregnancy were observed [25,26]. An elegant ecological analysis described by McNally et al suggested that environmental exposure and life style are associated with WT and soft tissue sarcomas [27]. Recently, a study from Ontario demonstrated a 30% decline in the incidence of WT after the folic acid flour fortification initiative [28].

Higher RB incidence rates were reported for other developing areas such as Malawi, Africa, and Colombia [4]. In Europe, North America, and Australia, RB corresponds to 2-4% of all tumours in infancy [29]. Data from the Automated Childhood Cancer Information System project showed that incidence rates were higher in northern Europe [30]. We observed a very high incidence rate of RB in Brazil, with more than 15 cases per million (0-4 years) similar to Mexico [10,19,31]. The higher RB incidence rate could correlate with deprivation. However, overall in the PBCRs, a significant correlation between the SEI of the location and RB incidence rates was not observed. Therefore, in Natal (SEI, 0.595), the RB incidence rates were high, whereas the RB incidence rate was the lowest in Curitiba (0.73). Clinically, RB occurs in two forms; a unilateral form that corresponds to 90% of non-inheritable (sporadic) RB, and a bilateral form that is inheritable or germinal, exemplifying the classical cases of a cancer with an inheritable genetic anomaly of tumour suppressor gene *RB1 *[5,32]. Detection of oncogenic papillomavirus (HPV) was described in different countries including Mexico and India [33,34]. Recently, two Brazilian studies investigating the presence HPV in paraffin-embedded tumour specimens demonstrated results ranging from 4.6% to 28%. Cases were ascertained in São Paulo and Campinas, both cities with referral centers for RB treatment [35,36].

Hepatoblastoma is the rarest embryonic tumour affecting children younger than the age of 5 years. The age-standardised incidence in European and America countries are 1.2 per million. There appears to be very little geographic variation world-wide, and the incidence rate is approximately 1 case per million children [23,37]. Because there are very few cases and they occurred in only 8 PBCR, caution should be used when interpreting the variation in the incidence of hepatoblastoma in Brazil.

## Conclusion

A limitation of the present study is that, firstly, underreported cases as well as misdiagnoses might occur in some areas. Other important limitations could be caused by the SES not being individually measured. Our study demonstrates distinct differences in incidence rates of embryonic tumours between Brazilian regions that are useful for prioritizing further research in terms of socioeconomic status and geographic variations.

## Competing interests

The authors declare that they have no competing interests.

## Authors' contributions

BDC, MSPO and JMOF reviewed, discussed, and analyzed the data. BDC, JMOF and MSPO contributed to the writing of the manuscript. All authors participated in the interpretation of results, read and approved the final manuscript.

## Pre-publication history

The pre-publication history for this paper can be accessed here:

http://www.biomedcentral.com/1471-2407/11/160/prepub
